# DNA methylation-based age prediction and telomere length in white blood cells and cumulus cells of infertile women with normal or poor response to ovarian stimulation

**DOI:** 10.18632/aging.101670

**Published:** 2018-12-08

**Authors:** Scott J. Morin, Xin Tao, Diego Marin, Yiping Zhan, Jessica Landis, Jenna Bedard, Richard T. Scott, Emre Seli

**Affiliations:** 1 IVIRMA New Jersey, Basking Ridge, NJ 07920, USA; 2 Sidney Kimmel Medical College, Thomas Jefferson University, Philadelphia, PA 19107, USA; 3 Foundation for Embryonic Competence, Basking Ridge, NJ 07920, USA; 4 Yale School of Medicine, New Haven, CT 06510, USA

**Keywords:** methylation, reproductive aging, epigenetics, epigenetic clock, telomere, cumulus cells

## Abstract

An algorithm assessing the methylation levels of 353 informative CpG sites in the human genome permits accurate prediction of the chronologic age of a subject. Interestingly, when there is discrepancy between the predicted age and chronologic age (age acceleration or “AgeAccel”), patients are at risk for morbidity and mortality. Identification of infertile patients at risk for accelerated reproductive senescence may permit preventative action. This study aimed to assess the accuracy of the “epigenetic clock” concept in reproductive age women undergoing fertility treatment by applying the age prediction algorithm in peripheral (white blood cells [WBCs]) and follicular somatic cells (cumulus cells [CCs]), and to identify whether women with premature reproductive aging (diminished ovarian reserve) were at risk of AgeAccel in their age prediction. Results indicated that the epigenetic algorithm accurately predicts age when applied to WBCs but not to CCs. The age prediction of CCs was substantially younger than chronologic age regardless of the patient’s age or response to stimulation. In addition, telomeres of CCs were significantly longer than that of WBCs. Our findings suggest that CCs do not demonstrate changes in methylome-predicted age or telomere-length in association with increasing female age or ovarian response to stimulation.

## Introduction

A growing number of contemporary women choose to delay parenthood, making ovarian aging an increasingly important reproductive health issue. In the United Sates, the number of women delivering their first child after the age of 35 increased from 1/100 in 1970 to 1/10 in 2016; the average age of first-time mothers increased from 21.4 to 26.6 during the same time period [1]. Within the context of infertility treatment, ovarian aging is often used to refer to the declining potential of ovaries to produce oocytes in adequate number or quality in response to controlled ovarian stimulation [2]. Patients who present with ovarian aging are commonly diagnosed with diminished ovarian reserve (DOR), and offered *in vitro* fertilization (IVF) using either their own eggs or donated eggs or embryos. The number of women undergoing IVF for DOR has been steadily increasing in the United States, where DOR accounted for 10% of assisted reproductive technology (ART) cycles in 2003, 14% in 2008, and 19% in 2015; the number of fresh IVF cycles using non-donor oocytes performed for women with diminished ovarian reserve almost doubled reaching over 13,000 cycles during the same time period [3]. Similarly, the number of women undergoing oocyte donation increased from 11,627 in 2003 to over 20,000 cycles in 2015 [3]. Despite the obvious significance of ovarian aging as a clinical entity, its pathophysiology remains poorly understood.

Recent work has demonstrated that the methylation profile of a wide range of somatic tissues can be used to accurately predict the chronologic age of humans. This concept, termed the “epigenetic clock”, was developed from a large dataset of methylation levels of >7,800 human tissue specimens from multiple longitudinal studies across various fields of medicine (such as the Women’s Health Initiative [WHI] and the Framingham Heart Study [FHS]) [4]. The predictive model was generated by performing an elastic net regression of methylation levels of thousands of CpG sites to determine which sites were most predictive of the age of the subject from which the DNA was derived. Published in 2013, Horvath described that the chronologic age of a subject could be predicted to within a median accuracy of 43 months by utilizing an algorithm derived from the 353 most predictive CpG sites across the genome.

This discovery was novel for multiple reasons. First, this was the first robust report demonstrating that the epigenome changes in a predictable pattern with age, a discovery that has widespread implications in the aging research. Second, the model was remarkably accurate in predicting chronological age across multiple different tissue types. Thus, if Horvath’s algorithm was applied to a white blood cell (WBC) that was only two days old, it was still capable of predicting whether it was derived from a 45-year-old or a 75-year-old patient with remarkable accuracy. The same algorithm could be used to predict the age of the same subject from a buccal swab or liver biopsy [5]. Likewise, additional factors such as risk for diseases or phenotypic trades have been recently incorporated into these algorithms so as to increase accuracy and clinical relevance [6].

Perhaps the most intriguing line of research that has resulted from this work, is evidence from multiple studies that subjects with a discrepancy between their epigenetic clock age prediction and their actual chronologic age appear to be at increased risk of morbidity. Data from a meta-analysis of multiple longitudinal cohort studies (including the WHI and FHS) suggest that patients with evidence of an accelerated epigenetic clock (AgeAccel; age prediction older than actual chronologic age) were at increased risk for all-cause mortality over subjects in which the algorithm accurately predicted age [7,8]. Among WHI patients in the study, AgeAccel of >10 years was associated with a 48% increased risk of death per annum [9].

These findings may have relevance to the study of female reproduction. Many infertile women demonstrate evidence of diminished ovarian reserve (DOR) or poor response to controlled ovarian stimulation (COS) consistent with accelerated follicular depletion, a finding associated with reproductive aging [10,11]. Interestingly, evaluation of the methylation profile of WBC, saliva, and buccal swabs of postmenopausal women demonstrate that earlier menopause is associated with AgeAccel in Horvath’s model [12]. However, because all samples were evaluated from postmenopausal women, it is unclear whether it is menopause that changes the methylation profile or if patients with AgeAccel prior to menopause could be predicted to exhibit premature reproductive aging. Furthermore, while the epigenetic clock has been evaluated in multiple reproductive tissues (endometrium, spermatozoa), it has not been tested in the cells most responsible for the decline ovarian steroid production associated with reproductive aging – follicular cells.

Telomere length (TL) is a known biomarker for biological aging and has been associated with the female reproductive lifespan [13,14]. Moreover, TL in WBCs has also been correlated with extrinsic epigenetic age acceleration (EEAA), a measure of DNA methylation derived from Horvath’s algorithm [15]. Therefore, it could be hypothesized that the relative amount of telomere DNA in follicular and white blood cells might as well differ in reproductive age women according to their age and ovarian response to stimulation. Indeed, a recent study reported that granulosa cells in patients with biochemical primary ovarian insufficiency (POI) possess significantly shorter telomeres than healthy controls, as was also the case for TL in leukocytes [16].

In the current study, we sought to evaluate 1) whether the epigenetic clock model of aging applied to reproductive age women accurately predicts chronologic age in peripheral somatic cells (WBCs) and follicular somatic cells (cumulus cells [CC]); 2) whether the age predicted by DNA methylation of these sites in WBCs or CCs changes in women with phenotypic evidence of altered progression of reproductive aging in the form of poor or unexpected good response to COS; and 3) whether the relative telomere length of CCs or WBCs is associated with patient’s age or response to hyperstimulation.

## RESULTS

### Patient characteristics

A total of 77 women were enrolled in the study (Groups A (young good responders; n=20), B (young poor responders; n=20), and C (older physiologic poor responders; n=20), and D (older unexpected good responders; n=17). Patient and cycle specific descriptive statistics for the younger groups (A and B) are provided in Table 1. Group B patients had lower anti-Müllerian hormone (AMH, a marker of ovarian reserve) and estradiol (E_2_) levels on day of trigger compared to group A. They also had fewer mature oocytes retrieved, fertilized oocytes at two pronuclei (2PN) stage, and clinically usable blastocysts. Descriptive statistics for the older reproductive age groups are provided in Table 2. While there was no difference in age, group C patients (older physiologic poor responders) had a greater body mass index (BMI) than Group D patients. Otherwise, the group C (older physiologic poor responders) had lower AMH and E_2_ levels, and fewer mature oocytes, 2PNs and blastocysts, compared to group D. Of note, two patients in Group C had no oocytes recovered at time of retrieval and thus CC methylation or telomere length analysis could not be performed.

**Table 1:** Patient and cycle characteristics for study participants in younger reproductive age group.

	**Group A** (young good responders)	**Group B** (young poor responders)	**P value**
n	20	20	**-**
Age (y)	32.3 (+/- 2.1)	33.2 (+/- 2.2)	0.16^a^
BMI (kg/m^2^)	25.6 (+/- 4.5)	26.5 (+/- 4.8)	0.56^a^
AMH	5.7 (3 – 12.6)	0.85 (0.3 – 2.2)	<0.001^b^
E_2_ on day of trigger	4903 (1972 - 7032)	1319.0 (669 – 2663)	<0.001^b^
No. of mature follicles (>15mm)	18 (15 – 32)	4 (3 – 5)	<0.001^b^
No. of metaphase II oocytes	17 (11 – 33)	4 (1 – 11)	<0.001^b^
No. of 2PN	15 (0 – 27)	3 (0 – 11)	<0.001^b^
No. of clinically usable blastocysts	7 (0 – 20)	2 (0 – 5)	<0.001^b^

^a^ Student’s *t*-test

^b^ Mann-Whitney *U* test

**Table 2:** Patient and cycle characteristics for study participants in older reproductive age group.

	**Group C** (older physiologic poor responders)	**Group D** (older unexpected good responders)	**P value**
n	20*	17	
Age (y)	41.9 (+/- 1.5)	41.8 (+/- 1.3)	0.86^a^
BMI (kg/m^2^)	27.9 (+/- 5.5)	24.3 (+/- 4.9)	0.046^a^
AMH	0.72 (0.13 – 1.2)	3.93 (1.8 – 17)	<0.001^b^
E_2_ on day of trigger	1096 (570 – 2501)	4222 (1655 – 6532)	<0.001^b^
No. of mature follicles (>15mm)	4 (1 – 5)	15 (11 – 27)	<0.001^b^
No. of metaphase II oocytes	3 (0 – 9)	13 (8 – 30)	<0.001^b^
No. of 2PN	3 (0 – 7)	11 (4 – 25)	<0.001^b^
No. of clinically usable blastocysts	1 (0 – 3)	4 (0 – 15)	<0.001^b^

*Two patients had no oocytes retrieved at time of oocyte retrieval

^a^ Student’s *t*-test

^b^ Mann-Whitney *U* test

### Evaluation of methylation-based age prediction using the Horvath model in WBCs and CCs of reproductive-age women

**Figure 1:**
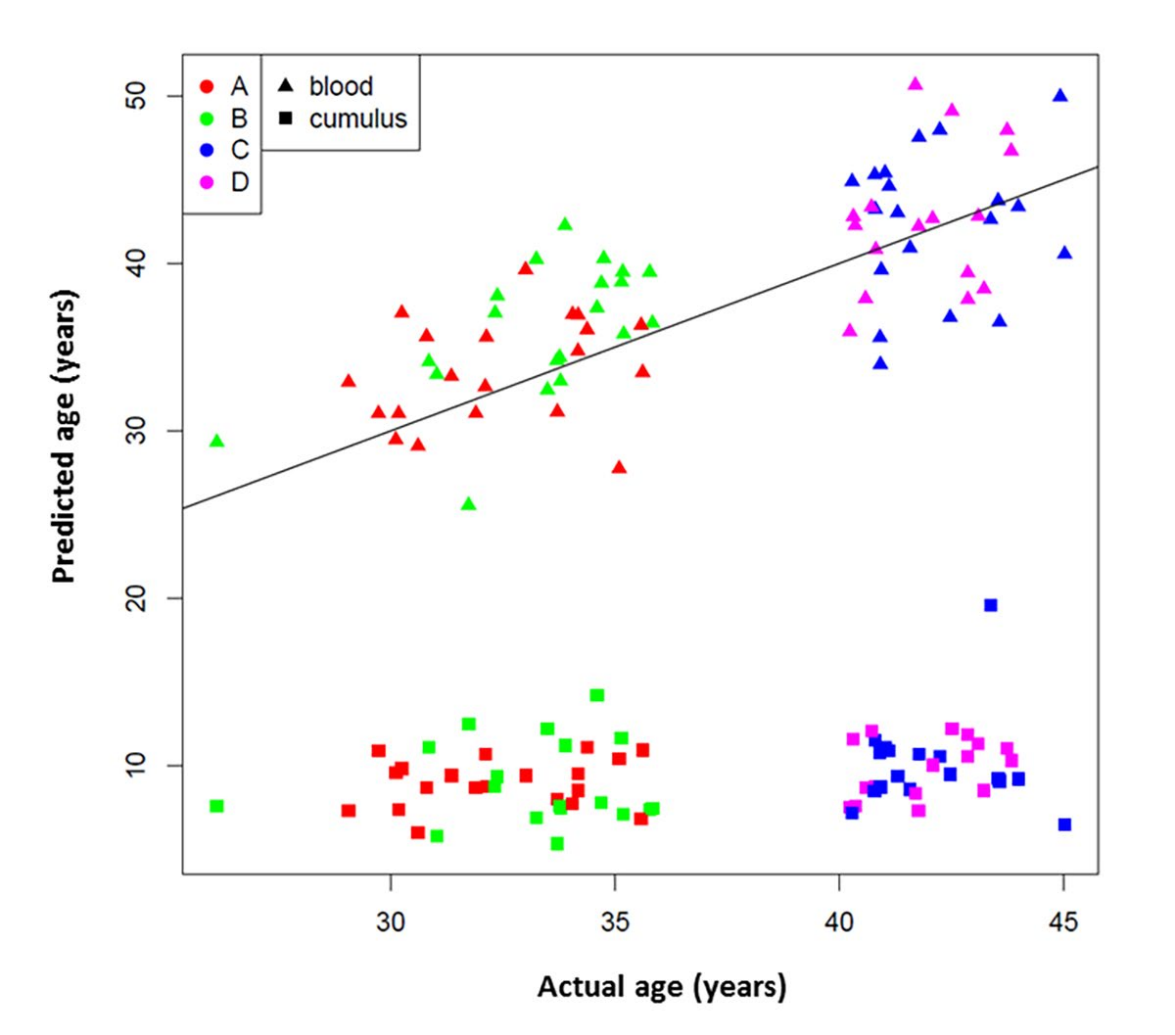
**353 CpG “epigenetic clock” age prediction in white blood cells and cumulus cells.** Black line indicates a perfect prediction of chronological age (y=x). Study groups are indicated with colors: Red A= <35 years old and good responder (≥15 mature follicles), green B= <35 years old and poor responder (≤5 mature follicles), blue C= >40 years old and poor responder (≤4 mature follicles) and magenta D= >40 years old and good responder (≥12 mature follicles). Tissue types are indicated by symbols.

The results of the age prediction analysis performed in WBCs and CCs, using the algorithm described by Horvath are depicted in Figure 1. Of note, three cumulus samples (two from group B and one from group C) were excluded from the analysis due to failure to pass QC standards. The age predicted by the 353 CpG site algorithm was consistent with the patient’s chronologic age in WBC samples (Figure 1). However, the overall DNA methylation level was significantly lower in CC samples compared to WBC samples from the same patient (p < 0.001). The predicted age in CCs was also substantially younger (Figure 1). The predicted age was unchanged whether the CC DNA was derived from patients in the younger age groups (groups A and B) or the older age groups (groups C and D); the predicted age was similar with an average age of 9.3 years old.

### Evaluation of whether ovarian response category alters methylation-based age prediction

When using a linear model that included age as a covariate, there was no association between the ovarian response category and age prediction of WBC methylation using the Horvath model on ANOVA (p = 0.12) (Figure 1). There was also no evidence of difference in the distribution of predicted ages when using CC-derived methylation levels on Wilcoxon rank sum (p = 0.36) (Figure 1). In other words, age prediction in CCs was not significantly altered according to the response category within age groups. However, it is notable that in 77.5% (31/40) of patients in Groups A and B, the WBC age prediction was older than true chronologic age. This was true in 85% (17/20) of Group B (young poor responders).

### Relative telomere length measurements in WBCs and CCs of reproductive-age women

**Figure 2:**
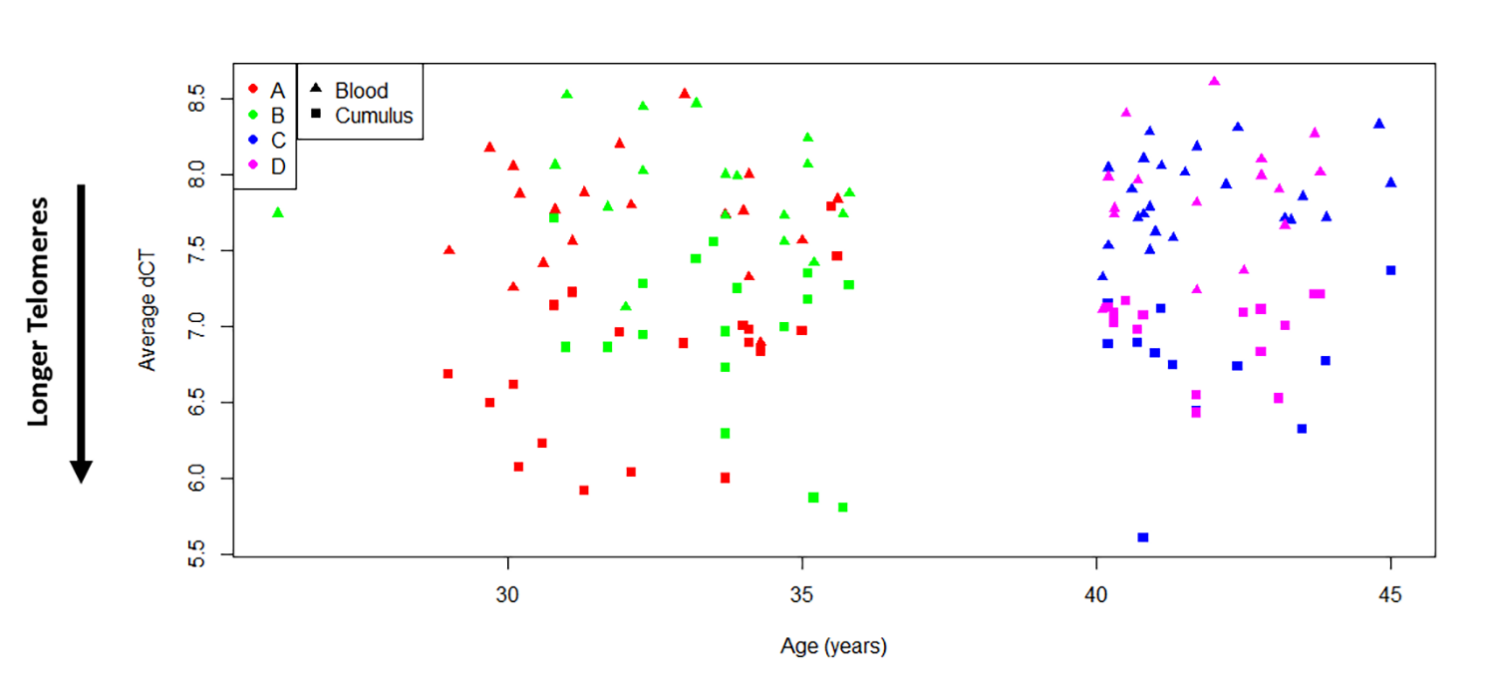
**Cumulus cells have longer telomeres than white blood cells.** Comparison of average relative telomere length of all study subjects indicates that ovarian cells present longer telomeres than white blood cells (t(138)=-14.46, p<0.0001). Tissues are indicated with symbols. Study groups are indicated with colors. A= <35 years old and good responder (≥15 mature follicles), B= <35 years old and poor responder (≤5 mature follicles), C= >40 years old and poor responder (≤4 mature follicles) and D= >40 years old and good responder (≥12 mature follicles). Average relative TL is reported as Average dCT values, which are inversely proportional to actual TL. Therefore, higher average dCTs indicate shorter telomeres.

Average relative TL was successfully measured in all WBC samples (77). However, genomic DNA from CCs was available for TL measurements in only 63 subjects after CC methylation assessment. Relative TL did not differ among the four study groups in both WBCs and CCs, and was therefore found not to be associated with patient’s age or ovarian response after stimulation (p=0.329; WBC F(3,73)= 0.9, p=0.4462) (Supplemental Figures 1 & 2). On the other hand, when comparing TL between tissues, CCs presented significantly longer telomeres than WBCs irrespective of the study group (p<0.0001, Figure 2). In addition, a paired analysis, which included only subjects with DNA available from both CCs and WBCs for TL measurements (59 subjects) also revealed that telomeres are significantly longer in CCs than in WBCs by an average of 2.16-fold change (p<0.0001, Supplemental Figure 3).

## DISCUSSION

These data demonstrate that an age prediction model according to the methylation level of 353 CpG sites in DNA derived from WBCs accurately predicts the chronologic age of reproductive age women pursuing assisted reproduction. However, the methylation profile of CCs does not provide an accurate age prediction according to the same model. Indeed, the algorithm’s age prediction was consistently younger in CCs regardless of the age of the subject, a finding that was in agreement with TL measurements, where CCs presented in average longer telomeres than WBCs. Furthermore, categorization of patients according to ovarian response did not appreciably change the age prediction in WBCs or CCs, neither was it associated with relative telomere DNA length.

This is the first prospective assessment of the “epigenetic clock” model of aging in women of reproductive age pursuing fertility treatment. While many reports across multiple medical disciplines have illustrated the accuracy of Horvath’s model for predicting chronologic age [17–19], and the association between accelerated epigenetic age (AgeAccel) and morbidity and mortality [7–9], reproductive medicine may provide a unique lens with which to evaluate usefulness of an epigenetic clock as a marker of general health and wellbeing. Indeed, reproductive longevity has been reported to correlate with overall longevity [20]. While establishing a robust model in this population will require significantly greater number of samples, further study could help illustrate whether the accelerated epigenetic profile described in women with earlier menopause [12] may already be evident during the reproductive years. If so, this information would be valuable for many reasons. Patients at risk for earlier menopause could benefit from lifestyle and medical interventions during their reproductive years to optimize cardiovascular and bone health as they face earlier cessation of ovarian steroid production. Furthermore, given that fertility declines long before perturbations in the menstrual cycle are evident, patients at risk of premature reproductive aging could take measures to ensure their family building plans are addressed in a timely fashion.

With the premise that a poor response to stimulation may represent the manifestation of premature reproductive aging in mind, we attempted to study whether patients with diminished ovarian reserve exhibited a methylation profile consistent with accelerated aging in somatic cells which had previously been demonstrated to fit the algorithm – WBCs. While there was no clear association evident, our classification strategy may have been insufficient to highlight subtle differences according to response phenotype. It is also possible that infertile patients in general (even those with evidence of a normal ovarian response) may demonstrate alterations in their epigenetic profile, such that the age prediction overlapped between groups. Indeed, while the age prediction model was accurate in WBCs within the margin of error difference observed in prior studies of the epigenetic clock (a median error of 3.6 years) [4], the majority of patients in the younger age group demonstrated an age prediction that was higher than their actual chronologic age, and this was more prominent in young poor responders. While this could represent an assay related or analytical artifact, future studies would be well served to use fertile controls and study whether infertility patients in general exhibit any evidence of accelerated aging according to the epigenetic clock model. While large numbers would be needed, this study design would help elucidate whether this model provides any predictive value for helping identify patients at risk for subfertility.

The ineffectiveness of the model when applied to CCs also raises interesting questions. While the epigenetic clock is accurate across many different tissue types, there are some tissues that do not produce the same predictable changes in methylation levels. In addition to CCs, breast and endometrial cells also do not fit the Horvath algorithm. It is possible that there may be a unifying mechanism responsible for explaining why these tissues – all of which respond to the cyclic hormonal changes of the menstrual cycle – do not exhibit the same epigenetic pattern of aging. Notably, unlike CCs, breast and endometrial tissues exhibit older than expected age predictions according to the epigenetic clock [4,21]. It is possible that different CpG sites may be altered in a predictable manner in these hormone responsive tissues. These sites may also be altered in infertile patients. Indeed, dysregulated epigenetic profiles have been described in women with endometriosis [22,23].

Alternatively, the young age prediction in CCs, consistent with young predictions seen in stem cells, could be the result of the communication between oocytes and CCs through gap junctions [24]. Indeed, oocyte-specific factors play key roles in granulosa/cumulus cell function, and targeted deletion of oocyte-specific genes may result in aberrant cumulus function associated with loss of gap junctions and transzonal processes [25–27]. Importantly, TL measurements were in agreement with the aforementioned results of no correlation of the epigenetic clock with ovarian response to hyperstimulation, and with the younger age prediction in CC compared to WBCs, since the former tissue type presented longer telomeres by an average fold change of 2.16. Attrition of telomeres has been extensively established to be correlated with cell aging, and was recently reported to be enhanced in follicular cells of patients with primary ovarian insufficiency [14,16]. Although the telomere theory of reproductive aging has mostly focused on the effect of shorter telomeres on meiotic disruption and higher risk of aneuploidies [28,29], it is also possible that the length of telomeres in follicular cells might play a role in oocyte maturation and the entire ovarian environment. As stated above, engagement in communication with the oocyte might regulate programmed aging by both epigenetic changes and telomere remodeling in these cells, in contrast to other peripheral tissues. At the same time, the expected correlation between subject’s age and telomere length was not found in either tissue type, probably because of a limited sensitivity of the TL assay to differentiate subtle changes in TL between patients.

Our findings demonstrate that an aging algorithm developed based on somatic cell methylome analysis fails to predict CC senescence or ovarian response to stimulation. While, this finding could be due to alternative methylation of CpG sites in CCs, longer TL observed in CCs compared to WBCs suggests a younger phenotype, possibly due to a continuing bidirectional communication with the oocyte. Further studies are needed to establish whether oocyte-CC communication affects age-related sites in the methylome, and whether an algorithm can be developed using alternative CpG sites in CCs that would predict patient age and/or ovarian response to stimulation.

## MATERIALS AND METHODS

### Design

All patients were recruited under Institutional Review Board approval during *in vitro* fertilization (IVF) cycles at Reproductive Medicine Associates of New Jersey (Basking Ridge, NJ) between February and July 2017. Each IVF cycle was managed by the patient’s physician per typical clinic protocol. Doses of gonadotropins and protocols were selected according to results of the patient’s precycle, ovarian reserve testing by anti-Müllerian hormone (AMH) and antral follicle count. Stimulation was achieved with injectable medications with follicle stimulating hormone (FSH) (recombinant or urinary) and luteinizing hormone (LH) activity (human menopausal gonadotropin or low dose human chorionic gonadotropin). Prevention of a premature LH surge prior to oocyte retrieval was achieved with either a gonadotropin releasing hormone (GnRH) antagonist on day 8 of the menstrual cycle or via pituitary downregulation with GnRH agonist. Doses of gonadotropins were adjusted according to response to stimulation (as determined by serial sonographic assessments of follicle growth and estradiol levels). The timing of administration of human chorionic gonadotropin or GnRH agonist trigger for final oocyte maturation occurred when the patient’s primary physician determined that a maximum number of follicles had reached the ideal size for retrieval of a mature oocyte (between 15 and 22mm in diameter). In general, this occurred when at least 2 follicles measured 18mm in diameter. Oocyte retrieval was performed 36 hours after administration of the trigger shot.

Recruitment occurred on day of trigger injection for final oocyte maturation. In order to analyze whether ovarian response to stimulation was associated with alterations in the epigenetic profile, four categories of patients were recruited. Group A consisted of women <35 years old with >14 mature follicles (at least 15mm on transvaginal ultrasound) (young good responders). A threshold of >14 mature follicles was selected as this represented the top tertile of patients in this age group in this clinic according to internal data. Group B consisted of women <35 years old with <5 mature follicles (at least 15mm) on day of trigger (young poor responders). A threshold of <5 mature follicles was selected as this represented the bottom 15^th^ percentile of patients in this age group according to internal data. Group C consisted of women >40 years old with <5 mature follicles (old physiologic poor responders) (bottom tertile). Group D consisted of women >40 years old with >12 follicles (old unexpected good responders) (top 15^th^ percentile).

On the day of oocyte retrieval, enrolled patients had a tube of peripheral blood drawn for evaluation of WBC methylation analysis. At time of oocyte retrieval, the follicular fluid of each enrolled patient was evaluated in a Falcon 1029 dish for evidence of cumulus oocyte complexes (COCs) per standard clinical routine. Identified COCs were isolated from cellular debris and blood and placed in a separate Falcon 1007 dish for washing. After all COCs were placed in the wash dish, they were passed in and out of a sterile, fire polished, glass Pasteur pipette three times to remove any debris that accompanied COCs into the wash dish. Cleaned COCs were then placed in a separate Falcon 1007 and excess cumulus cells were mechanically dissected from cumulus oocyte complexes with 27g needles. Oocytes were then moved to an organ well dish for continued culture, and the cut cumulus cells were pooled together for DNA isolation.

### Methylation analysis

Isolation of WBC DNA from peripheral blood samples was performed using the QIAsymphony kit (Qiagen, Redwood City, CA, USA). CCs DNA was purified using DNeasy blood and tissue kit (Qiagen, Redwood City, CA, USA). Bisulfite conversion was then performed using the Zymo EZ DNA methylation kit (Zymo Research, Irvine, CA, USA). The Illumina 850K DNA methylation EPIC array (San Diego, CA) was then utilized to measure DNA methylation levels throughout the genome [30,31].

### Methylome data analysis: evaluation of Horvath age prediction in reproductive-age women

To evaluate the age prediction by the Horvath algorithm, beta values were calculated using the intensities of methylated and unmethylated signals for each of the 353 informative CpG sites described by Horvath [4]. This analysis was performed separately for the WBC samples and the cumulus samples.

### Methylome data analysis: evaluation of whether ovarian response category alters age prediction

A linear model with female age as a covariate was used to evaluate the relationship between the ovarian response group and the age predicted by the Horvath algorithm with ANOVA. A Wilcoxon rank sum test was carried out for evaluating whether samples from poor response and normal response groups had the same distribution (Figure 2).

Data analysis was carried out with R version 3.4.0 (R Development core 2008). Beta value calculations, background correction and normalization by ssNoob [32], and data quality control were carried out with the minfy R package (version 1.24.0) [33]. A total of 18 control samples were included in the methylation analysis to assess for data normalization. Data quality control (QC) was also performed by inspecting intensity metrics and raw beta distributions.

### Relative Telomere Length Measurements

Relative TL was measured as previously described [29,34] by means of quantitative real-time PCR using isolated genomic DNA from both WBCs and CCs as starting material. PowerUp SYBR green Master Mix was used as detector (Applied Biosystems). qPCR quantification was performed in a 7900HT fast real time PCR system (Thermofisher Scientific). A Taqman Custom gene expression assay for the multicopy gene *Alu* (Assay ID: APPRKX3, Thermofisher Scientific) was used as endogenous control for normalization of TL measurements for each patient.

Four technical replicates for each subject and tissue type were established. Cycle threshold (Ct) values of the Tel assay (PCR reaction of *telc* and *telg* primers) and *Alu* assay were subtracted for each sample. Next, an average delta Ct (dCt) value was generated for each patient from all four technical replicates. Therefore, Ct values are inversely proportional to actual TL, where lower values indicate longer telomeres.

Statistical tests (Student’s t-test and ANOVA) for analysis of relative telomere length measurements were performed with the Analyse-it software for Microsoft Excel (version 2.20).

## SUPPLEMENTARY MATERIAL

Supplemental Figure 1

Supplemental Figure 2

Supplemental Figure 3
