# Supplementary material for: DNA methylation-based age prediction and telomere length in white blood cells and cumulus cells of infertile women with normal or poor response to ovarian stimulation
Source: Aging (Albany NY). 2018 Dec 8;10(12):3761–73. doi: 10.18632/aging.101670 (PMC6326671; doi:10.18632/aging.101670)
Supplement: Supplemental Figure 1 [file aging-10-101670-s001.pdf]

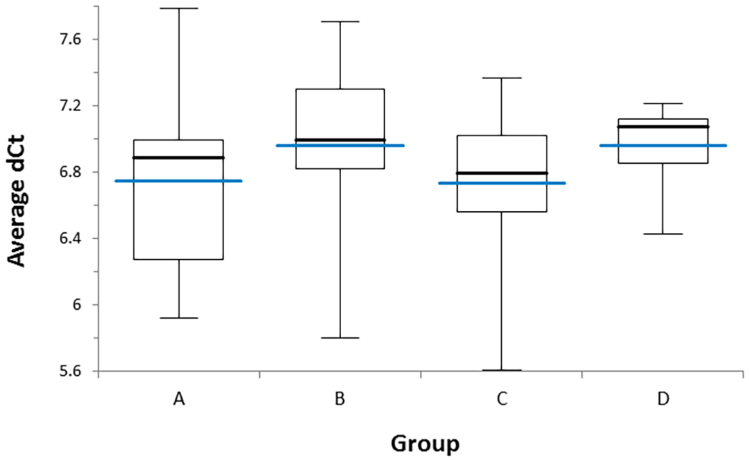

Supplemental Figure 1. Relative telomere length in cumulus cells is not associated with subject's age or ovarian response after controlled ovarian hyperstimulation. Distribution of average relative telomere length measurements among study groups (ANOVA: $F(3,59) = 1.17$, $p=0.329$). A= <35 years old and good responder (≥15 mature follicles), B= <35 years old and poor responder (≤5 mature follicles), C= >40 years old and poor responder (≤4 mature follicles) and D= >40 years old and good responder (≥12 mature follicles). Blue lines indicate mean values. Black lines indicate median. Average relative TL is reported as Average dCT values, which are inversely proportional to actual TL. Therefore higher average dCTs indicate shorter telomeres.
