# Supplementary material for: DNA methylation-based age prediction and telomere length in white blood cells and cumulus cells of infertile women with normal or poor response to ovarian stimulation
Source: Aging (Albany NY). 2018 Dec 8;10(12):3761–73. doi: 10.18632/aging.101670 (PMC6326671; doi:10.18632/aging.101670)
Supplement: Supplemental Figure 3 [file aging-10-101670-s003.pdf]

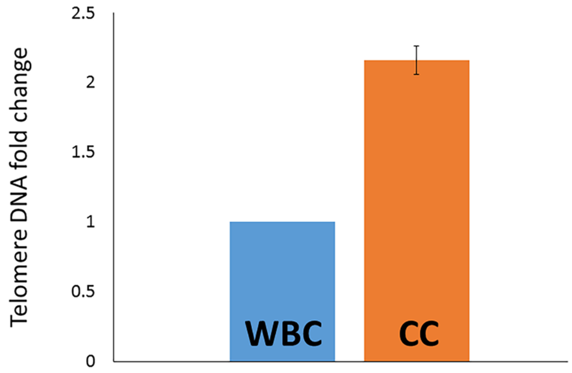

Supplemental Figure 3. Telomeres are significantly longer in cumulus cells than in white blood cells by an average 2.16-fold change. WBC: white blood cells, CC: cumulus cells. Paired analysis of average relative telomere length measurements in cumulus cells and leukocytes of 59 subjects with TL data of both tissues. WBC's TL was used as reference to calculate the fold change difference (Paired-t(116)= -14.05, p<0.0001) .
